# Molecular markers to assess short-term disease local recurrence in nasopharyngeal carcinoma

**DOI:** 10.3892/or.2015.3739

**Published:** 2015-01-20

**Authors:** TAO XU, BOJIN SU, CHUNHUA WANG, SUMEI WANG, HECHENG HUANG, YUNBAO PAN, DONGHUI WANG, WEIHONG WEI, FRANÇOIS X. CLARET, HUILING YANG

**Affiliations:** 1 Department of Pathophysiology, Zhongshan School of Medicine, Sun Yat-Sen University, Guangdong, Guangzhou 510600, P.R. China; 2 Department of Radiation Oncology, First People’s Hospital of Foshan, Guangdong, Foshan 528000, P.R. China; 3 Department of Systems Biology, The University of Texas MD Anderson Cancer Center, Houston, TX 77030, USA; 4 Department of Radiation Oncology, Cancer Center, Shantou Central Hospital, Guangdong, Shantou 515000, P.R. China; 5 Experimental Therapeutics Academic Program and Cancer Biology Program, The University of Texas Graduate School of Biomedical Sciences at Houston, Houston, TX 77030, USA

**Keywords:** nasopharyngeal carcinoma, recurrence, disease-free interval to recurrence

## Abstract

An important challenge in nasopharyngeal carcinoma (NPC) research is to develop effective predictors of tumor recurrence following treatment to determine whether immediate adjuvant therapy is necessary. We retrospectively analyzed archived specimens collected from 45 patients with paired samples of primary NPC (pNPC) and recurrent NPC (rNPC). Clinical samples were collected from the Cancer Center Databases of the First People’s Hospital of Foshan and Shantou Central Hospital (affiliates of Sun Yat-Sen University) between 2001 and 2012. Expression levels of phosphor-Stat3 (p-Stat3), signalosome complex subunit 5 (Jab1/Csn5), Akt1, C/EBP homologous protein (CHOP), Ki-67, and apoptosis were determined by immunohistochemistry in pNPC and rNPC samples from the same patients. Differences in these markers between the short-term interval to recurrence (ITR) group (ITR <18 months) and long-term ITR group (ITR ≥18 months) were further analyzed. In Cox’s regression analysis, the ITR was significantly associated as an independent-negative prognostic factor for overall survival (hazard ratio, 0.211; 95% confidence interval, 0.053–0.841; P=0.027). p-Stat3 was increased in the short-term ITR group (ITR <18 months) and tended to be lower in the long-term ITR group (ITR ≥18 months). In the short-term ITR group, nuclear Akt expression was significantly increased in paired rNPC (P=0.028). In the long-term ITR group, the expression of nuclear Jab1/Csn5 (P=0.047) and assessment of apoptosis measured with TdT-mediated dUTP nick end-labeling (TUNEL) (P=0.003) was significantly increased in paired rNPC. The results suggest that differences between short- and long-term ITR may predict outcome in rNPC. Furthermore, the overexpression of Jab1/Csn5 and Akt may contribute to the carcinogenesis of rNPC, and Akt seems to promote the progression of short-term ITR. Intra-individual changes of Jab1/Csn5, Akt, and TUNEL may help to identify short-term ITR.

## Introduction

Nasopharyngeal carcinoma (NPC), an Epstein-Barr virus (EBV)-associated cancer, is a major public health concern in Southeast Asia and Southern China, especially in the Guangdong province (1). Primary NPC (pNPC) has unique pathological and clinical characteristics, and radiotherapy with or without chemotherapy is the mainstream treatment. Although the 5-year survival of patients with NPC has steadily improved over the past three decades (2,3), overall 15–58% of patients experienced recurrence after radical radiotherapy in the era of conventional radiotherapy (4,5), and 13–22% have experienced recurrence in the era of intensity-modulated radiation therapy (6,7). In most patients with recurrence after complete remission following radical radiotherapy, the cancer returns within an average of 1.5 years, with local recurrences accounting for 70% of such cases (8,9). Recurrent NPC (rNPC) may be local, regional, or distant and is usually treated with radiation therapy and/or chemotherapy and occasionally with surgery. Retreatment for rNPC poses a critical challenge given its poor efficacy and serious toxicities (10).

Improved identification of prognostic factors by means of molecular testing may be useful in the diagnosis of diseases and their exact subtypes and may aid physicians in selecting individualized treatment, increasing the likelihood of local salvage. Several prognostic factors have been identified in recent years including recurrent tumor T stage, histologic type, patient age and disease-free interval to recurrence (ITR) (11–16). Of these factors, short-term ITR has been shown to correlate with poor outcome (14,15). Therefore, identification of molecular markers that may lead to an improved understanding of rNPC and to individualized treatment is imperative.

NPC arises from the mucosal epithelium of the nasopharynx, and recurrence is a complex multistep process involving several factors, such as maintenance of stem cells and epithelial-mesenchymal transition (17,18). Over 30 molecular markers have been studied as potential prognostic and/or predictive biomarkers for NPC, including markers associated with key cell functions such as cell proliferation, apoptosis, autophagy, and necrosis (19–22). However, the framework of rNPC has yet to be well established. The c-Jun activation domain-binding protein-1/constitutive photomorphogenic-9 (COP9) signalosome complex subunit 5 (Jab1/Csn5) has been shown to be involved in the pathogenesis of NPC and has been previously established as a tumor target by our group (23–25). Two additional critical signaling pathways, the phosphorylation of the signal transducer and activator of transcription 3 (Stat3) pathway and the phosphatidylinositol-3-kinase (PI3K)/Akt pathway, have also been identified in head and neck squamous cell carcinoma (HNSCC) and NPC (26–28). In addition, emerging evidence has shown that endoplasmic reticulum (ER) stress-activated unfolded protein response (UPR) has multiple roles in tumor development. C/EBP homologous protein (CHOP), also known as GADD153, is a critical protein that mediates ER stress-induced apoptosis (29,30). Therefore, we hypothesized that these factors contribute to the progression of rNPC.

However, the mechanistic link between ITR and outcome for rNPC has yet to be fully understood. In the present study, we collected paired pNPC and rNPC samples from 45 individuals from two institutions and compared the expression of Jab1/Csn5 (nuclear and cytoplasmic), phosphor-Stat3 (p-Stat3) (Tyr705), Akt (nuclear and cytoplasmic), CHOP, Ki-67, and terminal deoxynucleotidyl transferase (TdT)-mediated dUTP nick end-labeling (TUNEL) in the paired tumor tissues. Subsequently, we evaluated the differences between short- and long-term ITR.

## Materials and methods

### Ethics statement

All human tissues were collected using protocols approved by the Ethics Committee of the First People’s Hospital of Foshan and Shantou Central Hospital affiliated with Sun Yat-Sen University. Clinical and pathological data of patients were analyzed anonymously.

### Data collection and eligibility criteria

The cancer center databases at two institutions affiliated with Sun Yat-Sen University, the First People’s Hospital of Foshan and Shantou Central Hospital, were retrospectively reviewed for the period 2001–2012. The inclusion criteria for paired specimens of pNPC and rNPC were: i) the patient achieved complete remission of the primary tumor after radical radiotherapy (total dose of radiotherapy ≥70 Gy for the nasopharyngeal site); ii) rNPC was pathologically diagnosed in non-keratinizing cancer and was the same pathological type as pNPC; and iii) paired tissues were available from the primary tumor and the asynchronous local recurrence in the same patient. Two pathologists histologically confirmed all the biopsies. Clinical information including age, gender, and dates of diagnosis for pNPC and rNPC were obtained from the medical records. The clinical stage was designated according to the tumor, node, and metastasis (TNM) classification system of the American Joint Committee on Cancer/International Union Against Cancer (6th edition, 2002).

### Definitions of short- and long-term ITR

ITR was defined as the time between diagnosis of pNPC and rNPC. Although various time cutoffs for ITR have been evaluated previously, (including 12, 18, and 24 months), we defined short-term ITR as ITR <18 months and long-term ITR as ITR ≥18 months, following the studies of Teo *et al* and Oksüz *et al* (14,15).

### Human tissues and immunohistochemical analysis

Expression of Jab1/Csn5 (nuclear and cytoplasmic), p-Stat3, Akt (nuclear and cytoplasmic), CHOP, and Ki-67 was analyzed by immunohistochemical (IHC) techniques. An analysis was conducted of the paired tumor tissues used as well as the 4-μm continuous sections cut from the formalin-fixed, paraffin-embedded paired pNPC and rNPC specimens. IHC analyses of p-Stat3 (Tyr705) (M9C6, dilution 1:100; Cell Signaling Technology, Danvers, MA, USA), Jab1/Csn5 (Ab495, dilution 1:150; Abcam, Cambridge, UK), Akt1 (C73H10, dilution 1:200; Cell Signaling Technology), Ki-67 (M7240, dilution 1:80; Dako Cytoformation, Glostrup, Denmark), and CHOP (Ab27539, dilution 1:50; Abcam) were performed using the streptavidin-biotin-peroxidase complex technique. The procedure was as follows: conventional dewaxing hydration, 3% hydrogen peroxide treatment, then blocking by dropper solution for 20 min, antibody incubation at 4°C overnight, then goat anti-rabbit/goat anti-mouse IgG by dropper, incubation for 30 min at 37°C, followed by baths of diaminobenzidine and hematoxylin. Negative controls were analyzed along with each assay by replacing the primary antibody with phosphate-buffered saline.

### TUNEL

The TUNEL assay was used to determine cell apoptosis. Paraffin-embedded tissue sections (4-μm) were mounted on xylene-coated slides and dried at 37°C overnight. The sections were deparaffinized in xylene followed by sequential washes in graded ethanol and in phosphate-buffered saline. The samples were denatured by 15-min exposure to 20 μg/ml proteinase K at room temperature, and endogenous peroxidase activity was blocked with 3% hydrogen peroxide for 10 min. Apoptotically-fragmented cell DNA was identified by the TUNEL assay using the ApoTag kit (KeyGen, Nanjing, China). The samples were incubated with bovine TdT at 30 U/ml in a humid atmosphere at 37°C for 60 min, followed by exposure to anti-digoxigenin-labeled secondary antibody for 30 min at room temperature. After 2–10 min exposure to 0.05% diaminobenzidine in 0.02% hydrogen peroxide solution, the samples were counterstained with methyl green (0.5% in 0.1 M sodium citrate, pH 4.0), mounted, and dried.

### Scoring

The paired samples were independently evaluated by two investigators (W.C.H. and S.B.J.) without knowledge of the clinical information. The IHC results for the expression of Jab1/Csn5 (nuclear and cytoplasmic), p-Stat3, Akt (nuclear and cytoplasmic), CHOP, and Ki-67 were evaluated using a five-tiered semi-quantitative method. Five ×400 magnification fields for each section were scored as 0 for no membrane staining, 1 for weak staining, 2 for moderate, or 3 for strong staining. The extent of tumor cell membrane staining was scored from 0 to 5 (0%, <1%, 1–10%, 11–33%, 34–67% and >67%), and the staining intensity was scored from 0 to 3 (absent, weak, moderate and strong). Tumor proliferation and apoptosis were evaluated by semi-quantitative analysis using Ki-67 and TUNEL, respectively. The cells were counted under ×400 magnification fields, with positive cells defined as nuclei staining for Ki-67 (for the proliferation index) or cell staining for TUNEL (apoptotic index). Scores for each tumor were reported as the means of the percentage of positive cells per high-power field.

### Statistical analysis

SPSS 16.0 for Windows (SPSS, Inc., Chicago, IL, USA) was used for the statistical analysis. Stata 12.0 (StataCorp, College Station, TX, USA) was used to generate the forest plots. Expression of Jab1/Csn5 (nuclear and cytoplasmic), p-Stat3, Akt (nuclear and cytoplasmic), CHOP, Ki-67, and TUNEL was compared using the Wilcoxon signed-rank test in the paired pNPC and rNPC samples. Baseline values of these biomarkers were compared using the Mann-Whitney test. The Kaplan-Meier method was used to calculate cumulative survival. The log-rank test was used to compare the survival curves. The Cox proportional hazards model was used to analyze multiple prognostic factors for survival, including gender, age (<46 or ≥46 years), pT and pN category, pTNM staging, rT and rN category, rTNM staging, and ITR (<18 or ≥18 months). Two-tailed P-values <0.05 were considered statistically significant.

## Results

### Patient characteristics and survival

**Figure 1:**
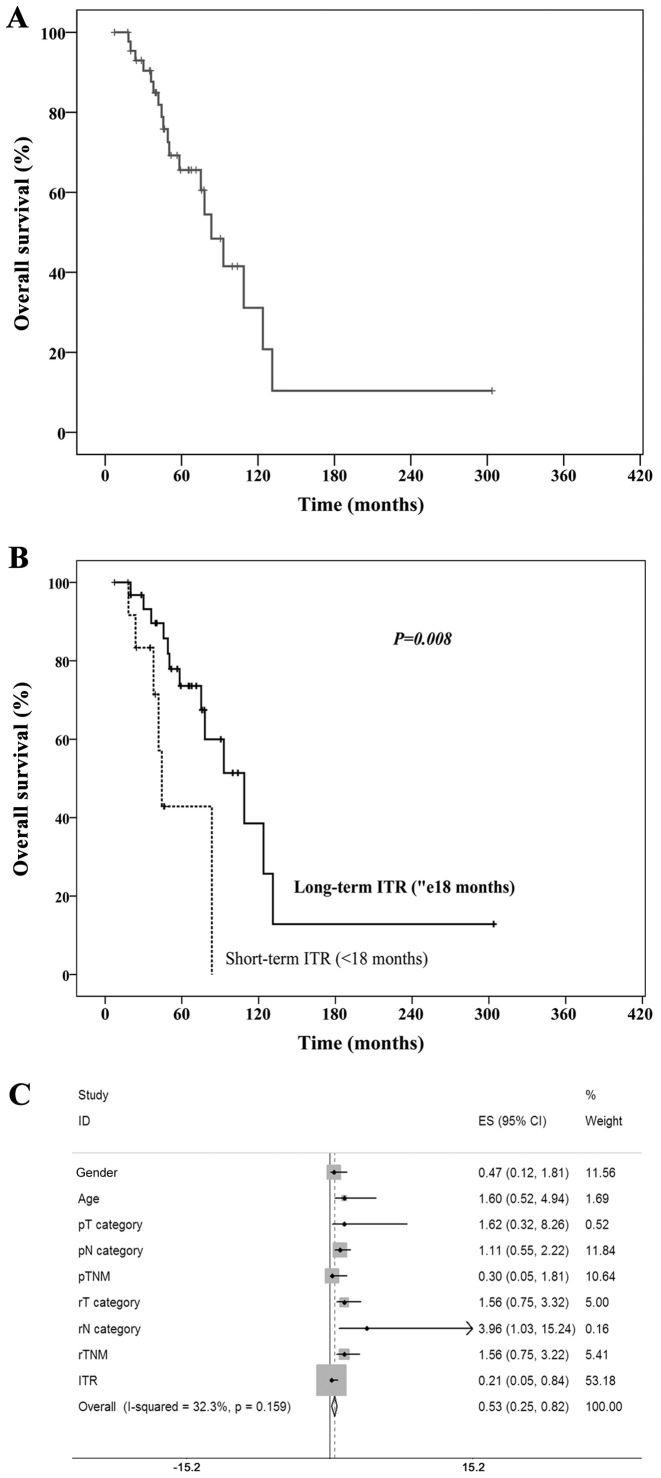
(A) Survival curves generated by the Kaplan-Meier analysis method to estimate overall survival. (B) Different survival curves of disease-free interval to recurrence (ITR) (< or ≥18 months) were compared with the log-rank test. (C) The Cox proportional hazards model was used to analyze the multiple prognostic factors for survival, including gender, age (<46 or ≥46), pT and pN category, pTNM staging, rT and rN category, rTNM staging, and ITR (<18 or ≥18 months). The forest plot was generated accordingly. TNM, tumor, node and metastasis.

Formalin-fixed, paraffin-embedded archive specimens were retrospectively available from 45 patients with paired biopsy-confirmed rNPC and pNPC samples. The baseline characteristics of these 45 rNPC patients are shown in Table I. All the patients were treated with radical radiotherapy at a median dose of 74 Gy (range, 70–76 Gy) for the nasopharyngeal tumor, and 80% of patients received combined chemotherapy. The 40 patients were treated again with radiotherapy and 5 patients underwent surgery. The median follow-up was 49 months (range, 7–147 months), and the median survival of the 45 patients with paired samples was 83.4 months (Fig. 1A).

### Confirmation of ITR as an independent prognostic factor

In the present study, the median ITR was 26 months (range, 5.0–281.5 months). There were 14 patients with ITR <18 months and 31 patients with ITR ≥18 months. For the 45 patients with paired tumor specimens, the median survivals of patients with short- and long-term ITR were 44.3 and 108.7 months (P=0.008), respectively (Fig. 1B). The multivariate analysis using Cox’s regression model revealed that ITR (<18 or ≥18 months) was the most important prognostic factor for overall survival (hazard ratio, 0.211; 95% confidence interval, 0.053–0.841; P=0.027) (Fig. 1C).

### Analysis of Jab1/Csn5, p-Stat3, Akt, and CHOP in paired pNPC and rNPC

**Figure 2:**
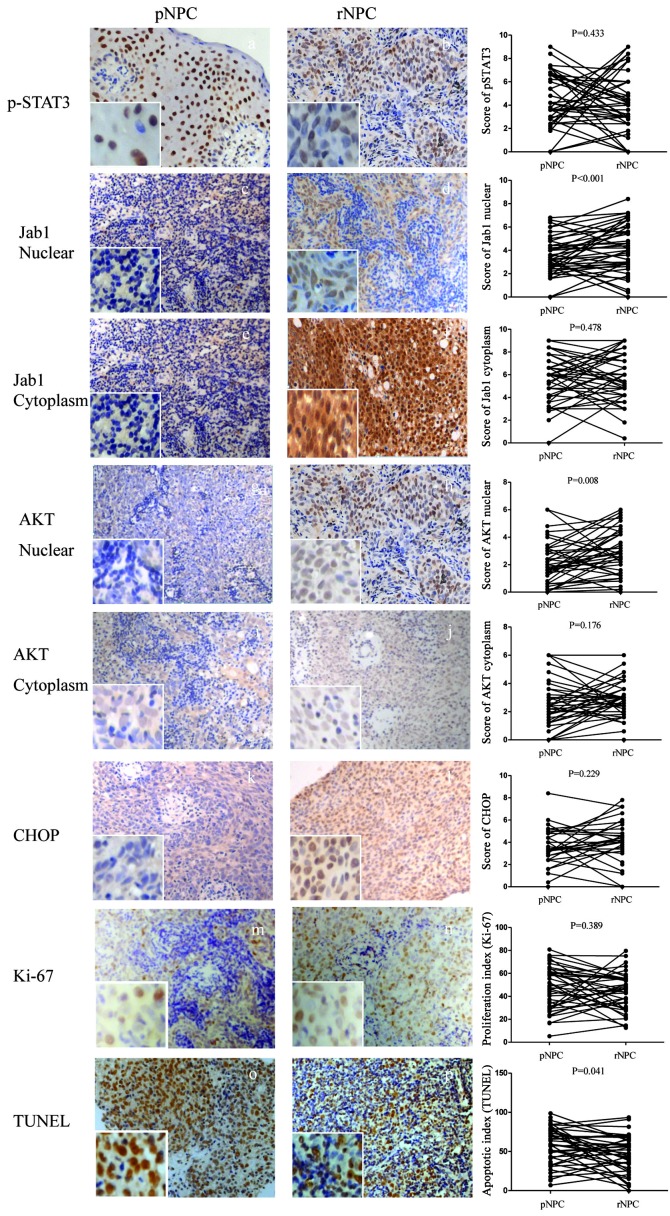
Immunohistochemical staining of (A and B) p-Stat3, (C and D) Jab1 nuclear, (E and F) Jab1 cytoplasm, (G and H) Akt nuclear, (I and J) Akt cytoplasm, (K and L) CHOP, and (M and N) Ki-67 in paired primary nasopharyngeal carcinoma (pNPC) and recurrent nasopharyngeal carcinoma (rNPC) samples. Apoptosis was measured by (O and P) TUNEL in paired pNPC and rNPC samples. Original magnification, ×200; insets ×400. p-Stat3, phospho-Stat3; CHOP, C/EBP homologous protein; TUNEL, TdT-mediated dUTP nick end-labeling.

Successful IHC evaluations of Jab1/Csn5, p-Stat3, Akt, and CHOP were performed in 44, 42, 41, and 29 specimens, respectively. The increased expression of nuclear Jab1/Csn5 (P<0.001) and nuclear Akt (P=0.008) was observed in rNPC compared with the paired pNPC specimens. There was no significant difference with p-Stat3 staining between paired pNPC and rNPC (P=0.433). In addition, we observed no change in cytoplasmic Jab1/Csn5 (P=0.478), cytoplasmic Akt (P=0.176), or CHOP (P=0.229) (Fig. 2).

### Subset analysis of Jab1/Csn5, p-Stat3, Akt, and CHOP status in short- and long-term ITR groups

**Figure 3:**
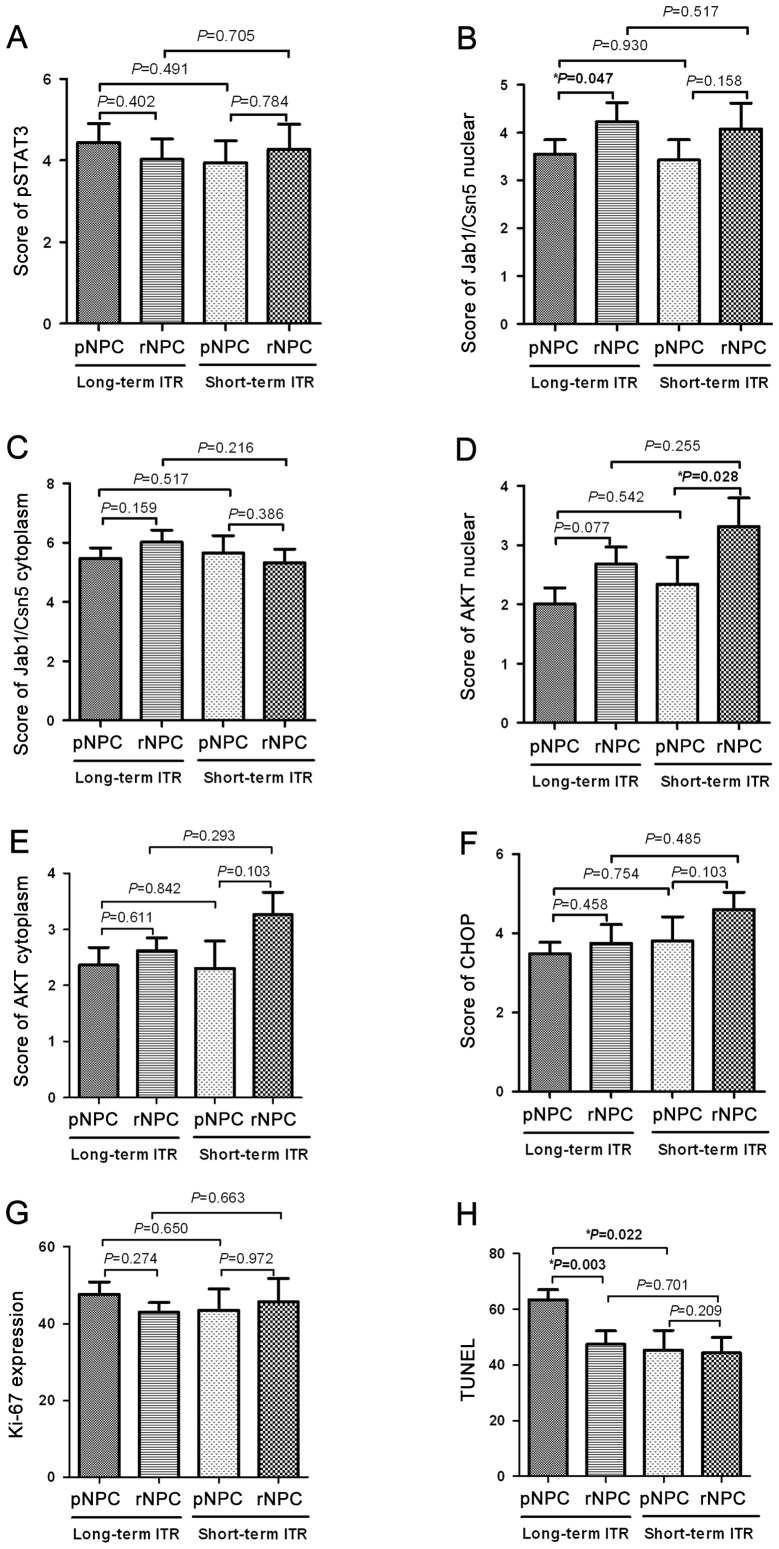
The expression of p-Stat3, Jab1 nuclear and cytoplasm, Akt nuclear and cytoplasm, CHOP, Ki-67, and TUNEL between long-term disease-free interval to recurrence (ITR) and short-term ITR in paired primary and recurrent nasopharyngeal carcinoma. (A) p-Stat3; (B) Jab1 nuclear; (C) Jab1 cytoplasm; (D) Akt nuclear; (E) Akt cytoplasm; (F) CHOP; (G) Ki-67; and (H) TUNEL. Stat3, phospho-Stat3; CHOP, C/EBP homologous protein; TUNEL, TdT-mediated dUTP nick end-labeling.

Results for Jab1/Csn5, p-Stat3, Akt, and CHOP status in the short-Cox regression multivariate analysis and long-term ITR groups are shown in Fig. 3. p-Stat3 increased in the short-term ITR group and decreased in the long-term ITR group but did not reach statistical significance. Compared with pNPC, Jab1/Csn5 nuclear expression significantly increased (P=0.047) in paired rNPC in the long-term ITR group. However, there was no significant difference in Jab1/Csn5 nuclear expression in the short-term ITR group (P=0.158). In contrast to Jab1/Csn5 status, an increased expression of Akt1 was associated with the short-term ITR group (P=0.028) in paired tissue samples. However, there was no significant difference in Jab1/Csn5 nuclear expression in the short-term ITR group (P=0.158). No significant difference in CHOP expression was observed in the short-term (P=0.103) or the long-term (P=0.458) ITR group.

### Analysis of Jab1/Csn5, p-Stat3, Akt, and CHOP status in pNPC and rNPC samples considering ITR subgroups

The pNPC samples in the long-term ITR group did not significantly differ in expression of Jab1/Csn5 nuclear (P=0.930), cytoplasmic Jab1/Csn5 (P=0.517), p-Stat3 (P=0.491), Akt nuclear (P=0.542), cytoplasmic Akt (P=0.842), and CHOP (P=0.754) compared with the short-term ITR group. Furthermore, no significant differences were observed in Jab1/Csn5 nuclear (P=0.517), cytoplasmic Jab1/Csn5 (P=0.216), p-Stat3 (P=0.705), Akt nuclear (P=0.255), cytoplasmic Akt (P=0.293), and CHOP (P=0.485) expression in the rNPC samples from the long-term ITR group, compared with rNPC samples from the short-term ITR group (Fig. 3).

### Significant apoptosis increased in pNPC between short- and long-term ITR

Proliferation and apoptosis were measured with Ki-67 and TUNEL, respectively. In the 45 paired tissue samples, Ki-67 staining did not differ significantly between pNPC and rNPC (P=0.389). However, we found by TUNEL assay that apoptosis was lower in rNPC compared with pNPC (P=0.041). In the subset analysis of the long-term ITR group, the Ki-67 results did not differ significantly between pNPC and rNPC samples (P=0.274). However, apoptosis measured by TUNEL was conspicuously lower in the rNPC samples than in the pNPC samples (P=0.003). We found that in the pNPC samples the baseline TUNEL results differed significantly between the short- and long-term ITR groups (median of 47.2 vs. 59.2%, P=0.022). Correlation analysis of Ki-67 and TUNEL indicated that there was no correlation between proliferation and apoptosis in the pNPC samples (P=0.566) or in the rNPC samples (P=0.890). Compared with the pNPC samples from the short-term ITR group, we only found a significant difference in TUNEL (P=0.022) in the pNPC samples from the long-term ITR group.

## Discussion

There are no generally acknowledged criteria for determining which patients are likely to develop rNPC, and identification of a subset of patients with worse clinical outcome has not been well established in rNPC. To define such criteria, prognostic factors for rNPC following radiotherapy treatment need to be determined. To the best of our knowledge, this is the first study on rNPC using paired initial and recurrent tumor specimens from the same patients to evaluate the significance of ITR. Thus, we analyzed the intra-individual changes between pNPC and rNPC of several biomarkers, especially between the short-term (<18 months) and long-term (≥18 months) ITR groups, and we further identified useful molecular markers for distinguishing the two groups.

An accurate definition of rNPC and the careful acquisition of study samples are crucial. Previous studies have been hampered by rNPC samples mixed with persistent tumor as well as by false-positive rNPC without pathologic confirmation (11–15). Although no standardized definition of rNPC exists, confirmation by biopsy is widely accepted as the ‘gold standard’. In most studies, however, only a small proportion of rNPC samples could be obtained and most patients had their disease diagnosed clinically, owing to the technical difficulty of obtaining biopsy specimens from sites of recurrence close to critical organs (10). In the present study, we analyzed 45 paired pNPC and rNPC samples from patients that presented at two different institutions located within an endemic area and covering a period of over a decade. We found that short-term ITR was closely associated with poor survival, and the Cox regression model supported ITR as an independent negative prognostic factor for rNPC (HR 0.211; 95% CI, 0.053–0.841; P=0.027).

Although rNPC is considered an incurable disease and patients need to receive palliative chemotherapy, several investigators nonetheless have observed long-term ITR patients with good outcomes (14,15). Heterogeneity of tumor cells and a biological mechanism involved in this process may be due to Jab1/Csn5, which positively regulates cell proliferation by inactivating p27, cyclin E, Smad 4/7, and p53 or by stabilizing hypoxia inducible factor (HIF-1α), which has been identified as a poor prognostic factor in several cancer types (31). Recently, we found that Jab1/Csn5 positively regulates the DNA repair gene *Rad51* and contributes to radiation resistance in NPC (23); Therefore, we hypothesized that Jab1/Csn5 participates in the process of recurrence after radical radiotherapy in NPC patients. The results of the present study show that nuclear Jab1/Csn5 was highly overexpressed in the paired rNPC tissues compared with pNPC samples. However, the subgroup analysis reveals that nuclear Jab1/Csn5 increased from pNPC to rNPC in the long-term ITR group. Therefore, another signaling pathway may exist in short-term ITR that facilitates tumor progression.

Stat3, an oncogenic transcriptional factor that is activated to initiate transcription of Stat3-targeted genes, is important in cell proliferation, invasion, and tumor formation. p-Stat3 is the main type that undergoes activation, and it has the potential to be an antitumor target (32,33). Overexpression of Stat3 has been observed in various cancer tissues including HNSCC (34) as well as in NPC (35). However, there is a contradiction between clinical outcomes and Stat3 activation. Although 67.5% of NPC samples were stained with p-Stat3 and Stat3 activation was positively associated with stage III and IV disease (28), Hsiao *et al* found that coactivation of constitutively activated Stat3 and Stat5 is associated with improved outcome. Thus, a geographic difference in Stat3 status may exist (36). The expression of p-Stat3 in rNPC has not been reported. In the present study, positive p-Stat3 expression was 84.09 and 80.00% in paired pNPC and rNPC, respectively. Pectasides *et al* deduced that Stat3 may play a dual role in HNSCC during different stages, depending on the genetic background (27). In the present study, we found that p-Stat3 tended to be greater in the short-term ITR group than in the long-term ITR group. Thus, p-Stat3 may play a dual role: in support of carcinogenesis in early events and of tumor suppression in late events.

The PI3K/Akt pathway is one of the most attractive signaling pathways for targeted therapy, especially given emerging evidence showing that this pathway plays a key role in EBV-induced disease, as demonstrated by increases in genomic instability, cell proliferation, and cytoskeleton dynamics and a decrease in apoptosis (37). Downstream activation of the Akt pathway is closely associated with radio-resistance (38), and a high expression of Akt1 has been associated with worse outcome after radiotherapy in HNSCC (39). Those findings support our results that Akt1 was highly expressed in rNPC compared with pNPC. Subgroup analysis shows that Akt1 was significantly elevated in the short-term ITR group (P=0.028) but not the long-term ITR group. We hypothesized that Akt1 is a key molecule in rNPC and that Akt activation contributes directly to the invasiveness of recurrent cancer cells and to a short-term ITR.

The imbalance between proliferation and apoptosis may determine the recurrence of a tumor and its recurrent stage. Aberrant apoptosis is required for rNPC development. In our study, proliferation remained active in recurrent tumors, compared with primary tumors, without significant change. However, apoptosis was suppressed in recurrent tumors. This was similar to the findings of Seong *et al* in paired colorectal cancer samples (40). We further analyzed the ITR subgroups and observed, contrary to our hypothesis, a significant decrease in apoptosis in the long-term ITR group. We found that the baseline TUNEL level was higher in the long-term ITR group than in the short-term ITR group. Hsiao *et al* found that ER stress/UPR may occur prior to EBV infection and trigger genetic changes in NPC carcinogenesis (29). CHOP expression is low under non-stressed conditions, but when ER stress-induced CHOP increases, it further triggers downstream proteins and induces cells to undergo apoptosis. We failed to observe any changes in CHOP expression between paired pNPC and rNPC samples.

In summary, results of the present biomarker study have shown that a biological mechanism is involved in the carcinogenesis of rNPC and that differences between short- and long-term ITR may predict outcome. Overexpression of Jab1/Csn5 and Akt may contribute to the carcinogenesis of rNPC, and Akt seems to promote the progression of short-term ITR. Intra-individual changes of Jab1/Csn5, Akt, and TUNEL may help to identify short-term ITR.

## Figures and Tables

**Table I:** Baseline characteristics of the patients with paired specimens of primary and recurrent nasopharyngeal carcinoma.

Characteristics	No. of cases (%)
Total patients	45 (100)
Age (years)	
<46	19 (42.2)
≥46	26 (57.8)
Gender	
Male	37 (82.2)
Female	8 (17.8)
pT category	
T1–2	13 (28.9)
T3–4	32 (71.1)
pN category	
N0–1	20 (44.4)
N2–3	25 (55.6)
pTNM	
II	5 (11.1)
III	30 (66.7)
IV	10 (22.2)
rT category	
T1–2	12 (26.7)
T3–4	33 (73.3)
rN category	
N^−^	39 (86.7)
N^+^	6 (13.3)
rTNM	
I	4 (8.9)
II	9 (20.0)
III	20 (44.4)
IV	12 (26.7)
Interval to recurrence (months)	
<18	14 (31.1)
≥18	31 (68.9)

pNPC, primary nasopharyngeal carcinoma; rNPC, recurrent nasopharyngeal carcinoma; ITR, interval to recurrence; TNM, tumor, node, and metastasis.
